# Clinical isolation, biofilm formation, and pathogenicity analysis of different species of the *Stephanoascus ciferrii* complex

**DOI:** 10.3389/fmicb.2025.1570952

**Published:** 2025-04-09

**Authors:** Shilan Xu, Baiyuan Fan, Shuo Gao, Jia Jia, Yan Zhang, Han Shen, Wanqing Zhou

**Affiliations:** ^1^Department of Laboratory Medicine, Nanjing Drum Tower Hospital, Affiliated Hospital of Medical School, Nanjing University, Nanjing, Jiangsu, China; ^2^School of Medicine, Jiangsu University, Zhenjiang, Jiangsu, China

**Keywords:** *Stephanoascus ciferrii* complex, *Candida allociferrii*, *Candida mucifera*, *Galleria mellonella* larvae, biofilm, pathogenicity, antifungal treatment, drug resistance

## Abstract

The *Stephanoascus ciferrii* complex, comprising *Stephanoascus ciferrii, Candida allociferrii*, and *Candida mucifera*, is an emerging fungal pathogen with increasing isolation rates and antifungal resistance. However, detailed information about clinical isolation rates and pathogenicity comparisons among the three species are lacking. In order to fill in this information gap, this study aimed to investigate and compare the clinical isolation rates and pathogenicity of the three species. Twenty-seven *S. ciferrii* complex strains isolated from the secretion specimens of patients admitted to Nanjing Drum Tower Hospital between 2012 and 2023 were included. According to the results of ITS sequencing, there were 15 strains of *S. ciferrii*, 7 strains of *C. allociferrii*, and 5 strains of *C. mucifera*. Antifungal susceptibility testing demonstrated that the *S. ciferrii* complex exhibited high MICs against azole antifungal agents, particularly fluconazole, while it showed lower MICs against echinocandins. *S. ciferrii* displayed higher MICs against caspofungin than *C. allociferrii* (*P* < 0.05). The results of biofilm quantification using crystal violet staining indicated *C. allociferrii* exhibited stronger biofilm-forming ability than *S. ciferrii* in RPMI-1640 medium (*P* < 0.05), but there was no significant difference between *C. allociferrii* and *C. mucifera* or between *S. ciferrii* and *C. mucifera*. The results were similar with the metabolic activity by using XTT assay. The *G. mellonella* larvae infection experiments revealed that the survival rates of larvae infected by strains of the *S. ciferrii* complex were 60%, 50%, and 48% at 24 h, 48 h, and 72 h, respectively. Furthermore, the *G. mellonella* larvae lethality caused by *C. allociferrii* and *C. mucifera* were significantly higher than that caused by *S. ciferrii* (*P* < 0.001). This study is the first to describe and compare the pathogenicity and biofilm formation ability of the three species of *S. ciferrii* complex in the clinical context. Our research reveals the high prevalence of *S. ciferrii* in the complex and elucidates the correlation between fungal drug resistance, biofilm formation, and virulence, thus providing essential empirical evidence for further study of the clinical pathogenic characteristics of each species in the complex and treatment strategies.

## 1 Introduction

The global incidence of invasive fungal diseases is on the rise, with a significant increase in both prevalence and mortality rates (Soriano et al., 2023; Xiao et al., 2018). Of particular concern is the global rise of ascomycetous yeast infections, such as the emergence of *Candida auris*, with documented increases in infections and high rates of antifungal resistance (Chowdhary et al., 2023). However, there are also epidemiological data showing a shift toward non-*Candida* infections and the emergence of drug-resistant strains (Soriano et al., 2023; Xiao et al., 2018). Common yeasts such as *Candida albicans* and *Candida glabrata* have been extensively researched. However, despite the significant rise in the incidence of rare yeast infections, mirroring the broader trend of increasing fungal infections globally (Pérez-Hansen et al., 2019), there is much more research required on non-*Candida* yeast infections. One of these rare pathogenic yeast species is *Stephanoascus ciferrii*, an infrequent clinical fungus that was first identified by Kreger-van Rij in 1965 (Kreger-Van Rij, 1965). In 2001, Kumiko Ueda-Nishimura and Kozaburo Mikata introduced the concept of the *Stephanoascus ciferrii* complex based on intronic analysis of its *18S* rDNA (Kumiko and Kozaburo, 2002). The *S. ciferrii* complex, a heterothallic ascomycetous fungus, comprises *S. ciferrii, Candida allociferrii*, and *Candida mucifera* (Kumiko and Kozaburo, 2002). The *S. ciferrii* complex can cause infections at multiple sites, including the ear canal, skin, eyes, abdomen, and bloodstream (Andrew et al., 2013; Cosio et al., 2024). This complex, which has long been considered a rare yeast, has been increasingly identified in clinical settings and can cause both superficial and invasive infections that result in serious consequences (Bansal et al., 2021; Demiray et al., 2015). As an opportunistic causative agent, the *S. ciferrii* complex is typically non-pathogenic or causes only superficial infections in immunocompetent individuals. However, in immunocompromised patients, severe outcomes are possible, including chronic otitis media, bloodstream infections such as fungemia, severe pulmonary infections, and other systemic fungal diseases. These conditions are particularly concerning as they can affect various parts of the body and may be life-threatening (Bansal et al., 2021; Byrd et al., 2018; Gunsilius et al., 2001).

Recently, concerns regarding the pathogenicity and drug resistance of the *S. ciferrii* complex have gained prominence in clinical practice (Agin et al., 2011; Borman et al., 2019). A particularly problematic observation is that the majority of *S. ciferrii* complex isolates exhibit high resistance to fluconazole, a commonly prescribed antifungal agent, thus complicating treatment efforts (Bansal et al., 2021; Agin et al., 2011; Gunsilius et al., 2001). Furthermore, the occurrence of lesions predominantly in the ear canal, with a prevalence rate exceeding those at other anatomical sites, may result in poorer clinical outcomes (Cheng et al., 2004).

Taxonomic studies have shown that there are three species in the *Stephanoascus ciferrii* complex (Guo et al., 2021). In 2021, Guo et al. employed diverse methodologies to pinpoint 28 strains within the *S. ciferrii* complex at the species level. Their analysis demonstrated that only internal transcribed spacer (ITS) sequencing could effectively differentiate species within the complex, yet as for other conventional methods, such as biochemical analysis and mass spectrometry, does not allow for comprehensive species identification. Moreover, despite predominantly similar antifungal susceptibility profiles among the three species, notable differences still existed. Unfortunately, despite the increasing clinical significance of this complex, most studies focus on strains matching the known features of the complex, and current experimental evidence regarding pathogenic disparities between members of the *S. ciferrii* complex and therapeutic guidelines for the management of these infections remain inadequate. Overall, there is a clear need for further in-depth research into *S. ciferrii* complex infections, particularly the features and differences between species within the complex in terms of antifungal drug resistance, biofilm formation ability, and pathogenicity.

The present study aims to fill in the research gap discussed above by elucidating the pathogenic discrepancies within the *S. ciferrii* complex at the species level and exploring new strategies for antifungal therapy. We collected and identified 27 strains of the *S. ciferrii* complex isolated from Nanjing Drum Tower Hospital by using VITEK 2, mass spectrometry, and ITS. Furthermore, we performed antifungal susceptibility testing and biofilm formation assays to explore the properties of the three known species that form the complex. The pathogenicity of the *S. ciferrii* complex was further assessed through an animal infection model. This study highlights the distinct pathogenic characteristics of the three species, thus providing innovative *in vivo* experimental evidence for diagnosing and treating infections caused by this complex. To sum, the research offers valuable insights into the identification of species, biofilm formation processes, pathogenic properties, and novel treatment strategies in relation to the *S. ciferrii* complex from a species-specific perspective.

## 2 Materials and methods

### 2.1 Fungal strains

Twenty-seven *S. ciferrii* complex strains isolated from clinical secretion specimens obtained from patients admitted to Nanjing Drum Tower Hospital between 2012 and 2023 were included in this study. The strains were initially identified using the VITEK 2 system (bioMérieux, France) and matrix-assisted laser desorption ionization-time of flight mass spectrometry (MALDI-TOF MS) (bioMérieux, France), and the results were subsequently confirmed through ITS sequencing. Clinical data pertaining to infections caused by the *S. ciferrii* complex were collected and analyzed. In this study, the 27 strains of the *S. ciferrii* complex were isolated from patients upon initial admission. All of strains were collected before medication and then stored in a freezer at−80 degree celsius from 2012. *Candida parapsilosis* ATCC 22019 and *Candida krusei* ATCC 6258 were used as controls, as these species are commonly utilized as control strains to ensure the accuracy and reliability of antifungal susceptibility testing.

### 2.2 Culture and morphology identification

*S. ciferrii* complex strains were inoculated onto blood agar plate (BAP) (ThermoFisher Scientific, Cleveland, OH, USA), Sabouraud dextrose agar (SDA) (Zhengzhou Biocell Biotechnology, China), and CHROM agar (Shanghai Kemajia Mircrobiotec, China) and incubated at 35°C for 48 h. Colony morphology was observed daily, and micromorphology was examined using an optical microscope at 1,000 × magnification after Gram staining.

### 2.3 Species identification with VITEK 2

The VITEK 2 Compact YST test kit (bioMérieux, France) was utilized to identify the *S. ciferrii* complex strains based on their biochemical reaction characteristics, following the manufacturer's instructions. After incubation on SDA plates for 48 h at 35°C, single colonies were collected and suspended in saline (0.45% NaCl) to create a 2.0 McFarland yeast suspension. Subsequently, the strains were identified using the VITEK 2 Compact system.

### 2.4 Species identification with MALDI-TOF MS

MALDI-TOF MS was utilized for strain identification based on protein composition. All operations and conditions were in adherence with the manufacturer's guidelines. After incubation on SDA for 48 h at 35°C, single colonies were selected and placed on individual target plates. Each colony was mixed with 1 μL of VITEK MS-FA (containing 25% formic acid, supplied by bioMérieux, France) to induce lysis of the yeast cell walls. Once the mixture had dried, 1 μL of α-cyano-4-hydroxycinnamic acid, supplied by bioMérieux (France), was added to the spot. The target spot was then prepared for MALDI-TOF MS analysis, which was conducted using VITEK MS. An *Escherichia coli* reference strain (ATCC 8739) was used as a calibration control.

### 2.5 ITS sequencing

Colonies of *S. ciferrii* complex strains grown on SDA at 35°C for 48 h were selected to prepare yeast suspensions as templates for nucleic acid extraction and amplification of the ITS region. DNA extraction was performed using the Ezuop Column Fungi Genomic DNA Purification Kit (Sangon Biotech, Shanghai, China), and all procedures were performed according to the manufacturer's instructions. The ITS region DNA was amplified using the universal primers ITS1 (5′-TCCGTAGGTGAACCTCGGG-3′) and ITS4 (5′-TCCTCCGCTTATTGATATGC-3′), which are commonly used for amplification of the entire ITS region (Guo et al., 2021). The PCR amplification conditions were as follows: 1 cycle at 95°C for 10 min; 40 cycles at 95°C for 30 s, 56°C for 30 s, and 72°C for 45 s; and 1 cycle at 72°C for 10 min. The PCR products were quantified using agarose gel electrophoresis and then sequenced using the gold-standard Sanger sequencing technology, known for its high accuracy and reliability, provided by Sangon Biotech, Shanghai, China. Sequence similarity was determined using the BLAST program from the National Center for Biotechnology Information (http://www.ncbi.nlm.nih.gov/BLAST).

### 2.6 Antifungal susceptibility testing

Sensititre YeastOne™ YO10 (ThermoFisher Scientific, Cleveland, OH, USA) was employed for antifungal susceptibility testing in accordance with the manufacturer's guidelines. This method has similar performance to the CLSI M38-A2 method for detecting fungal drug sensitivity. Following incubation, selected colonies were used to prepare a yeast suspension matching the 0.5 McFarland turbidity standard. A 20 μL aliquot of the suspension was transferred to 11 mL of YeastOne broth. After thorough mixing, 100 μL of the mixture was added to each well of the plate, which was then sealed. After incubation for 48 h at 35°C, results were recorded according to the manufacturer's instructions. The minimum inhibitory concentration (MIC) was determined as the lowest concentration resulting in 50% inhibition of growth compared to the growth of the control. *C*. *parapsilosis* ATCC 22019 and *C*. *krusei* ATCC 6258 were included in each assessment as quality controls.

### 2.7 Cystal violet assay

Biofilm formation ability of the *S. ciferrii* complex was evaluated using the microtiter plate method, as previously described (Gao et al., 2024). The strains were first grown for 48 h at 35°C on a yeast extract peptone dextrose (YPD) agar plate, adjusted to a concentration of 1 McFarland, and then seeded at a concentration of 10^6^ CFU/mL in RPIM 1640 or YPD medium. A 200 μL aliquot of the inoculum solution was added to each well of a microplate, which was then incubated at 35°C for 48 h. After incubation, the solution was discarded, and the plate was washed with saline (0.9%) three times and dried. The plate was then stained with 100 μL of 0.5% crystal violet (Beyotime Biotechnology, China) for 15 min and washed with saline (0.9%) three times. Following this, 200 μL of absolute ethanol was added, and absorbance was measured at OD570_nm_ using a MB-580 microplate reader (HEALES, China) (Sasani et al., 2021a). Each assay was performed with three replicates for each strain. The sterile culture medium was set as the blank control, while *Staphylococcus epidermidis* ATCC 12228 and ATCC 35984 served as negative and positive controls, respectively (Gao et al., 2024).

### 2.8 XTT assay

The XTT (Sigma, USA) solution was prepared at a concentration of 0.5 mg/mL using PBS (Marton et al., 2023). The solutions were filter sterilized through a 0.22-μm-pore-size filter. Menadione (Sigma, USA) was dissolved in DMSO and freshly prepared before use, an XTT/menadione solution was prepared to achieve a final concentration of 1 μmol/L of menadione. After incubation, the biofilms were washed three times with 200 μL of PBS, and then 100 μL of the XTT/Menadione solution was added to each of the wells. The microtiter plate was then incubated in the dark for 2 h at 37°C. Then, a colorimetric change in the XTT assay was measured at OD450_nm_ using a MB-580 microplate reader (HEALES, China).

### 2.9 *Galleria mellonella* larvae infection assays

*Galleria mellonella* larvae (Tianjin Huiyude Company, China), weighing 0.25–0.35 g, were used to assess the pathogenicity of *S. ciferrii* complex strains, as previously described (Cotter et al., 2000). Five strains of each species were selected and inoculated on SDA for 48 h at 35°C. Single colonies were blended with saline to prepare a yeast suspension at a concentration of 1 × 10^8^ cells/mL for inoculation. Prior to injection, the larvae's injection sites were sterilized with povidone-iodine solution. *G. mellonella* larvae, in groups of ten, were injected with 10 μL of yeast suspension through the last proleg using 25 μL gas-phase microliter syringes with a needle diameter of 0.5 mm. There were three experimental groups based on the three species of the *S. ciferrii* complex. Each group was further divided into five subgroups, each receiving yeast suspensions from different strains of the same species, covering a total of 15 strains. Additionally, two control groups were included: the blank control group comprising larvae that were untouched and maintained at the same temperature as the test larvae and the normal saline (NS) control group comprising larvae whose proleg was injected with 10 μL sterile saline. The larvae were placed in sterile petri dishes and incubated in the dark at 37°C. Their mortality rates were monitored daily over a 96-h period. Death was assessed based on lack of movement in response to stimuli combined with discoloration of the cuticle.

### 2.10 Statistical analysis

The data were analyzed using GraphPad, version 10.0 (GraphPad Software, United States). Quantitative data were analyzed with the *t*-test or the Mann-Whitney *U*-test, if they were normally or non-normally distributed, respectively. The Gehan-Breslow-Wilcoxon test was used to statistically analyze the survival curves of infected *G. mellonella* larvae. Statistical significance was set at *P* < 0.05.

## 3 Results

### 3.1 Identification and morphology of the *S. ciferrii* complex

By comparing the ITS sequences with reference sequences deposited in the NCBI database, 27 clinical strains of the *S. ciferrii* complex were identified and classified as 15 strains of *S. ciferrii* (55.56%) (GCA_030573635.1), 7 strains of *C. allociferrii* (25.93%) (CBS5156), and 5 strains of *C. mucifera* (18.52%) (CBS7409) (Table 1). The identification rate with MALDI-TOF MS was 51.85% (14/27): with this method, it was possible to successfully identify 80% (12/15) of the *S. ciferrii* strains, 14.29% (1/7) of the *C. allociferrii* strains, and 20% (1/5) of the *C. mucifera* strains. Additionally, the VITEK 2 Compact system confirmed that all 27 strains belonged to the *S. ciferrii* complex.

**Table 1:** Identification profiles of the 27 strains of *Stephanoascus ciferrii* complex.

**Number**	**ITS sequencing**	**VITEK compact**	**VITEK MS**
S-1	*Stephanoascus c*	*Candida ciferrii* (*Stephanoascus ciferrii*)	*Candia ciferri*
S-2	*Candida mucifera*		No result
S-3	*Stephanoascus ciferrii*		*Candia ciferri*
S-4	*Candida allociferrii*		*Candia ciferri*
S-5	*Candida allociferrii*		No result
S-6	*Stephanoascus ciferrii*		*Candia ciferri*
S-7	*Candida mucifera*		*Candia ciferri*
S-8	*Stephanoascus ciferrii*		*Candia ciferri*
S-9	*Candida allociferrii*		No result
S-10	*Candida mucifera*		No result
S-11	*Stephanoascus ciferrii*		No result
S-12	*Stephanoascus ciferrii*		*Candia ciferri*
S-13	*Stephanoascus ciferrii*		No result
S-14	*Stephanoascus ciferrii*		*Candia ciferri*
S-15	*Candida allociferrii*		No result
S-16	*Candida mucifera*		No result
S-17	*Candida mucifera*		No result
S-18	*Stephanoascus ciferrii*		No result
S-19	*Stephanoascus ciferrii*		*Candia ciferri*
S-20	*Stephanoascus ciferrii*		*Candia ciferri*
S-21	*Candida allociferrii*		No result
S-22	*Stephanoascus ciferrii*		*Candia ciferri*
S-23	*Candida allociferrii*		No result
S-24	*Stephanoascus ciferrii*		*Candia ciferri*
S-25	*Candida allociferrii*		No result
S-26	*Stephanoascus ciferrii*		*Candia ciferri*
S-27	*Stephanoascus ciferrii*		*Candia ciferri*

The strains were cultured on BAP, CHROM agar, and SDA at 35°C to induce colony growth. On BAP, the colonies appeared white, small, and needle-like after 24 h, turning faint yellow and cauliflower-like while becoming embedded in the agar. On CHROM agar, the colonies maintained a small, needle-like appearance, with most remaining white and a minority transitioning to aquamarine blue after 24 h; after 48 h, the colonies enlarged and exhibited gyri-like structures, with a significant portion developing aquamarine blue coloration. On SDA plates, the colonies were tiny and white after 24 h and later became dry, hard, and cauliflower-like. Notably, there was little morphological variation among the species, although the blue-green colonies on the CHROM agar may have been specific to and may aid in the recognition of *S. ciferrii*.

### 3.2 Susceptibility of the *S. ciferrii* complex to antifungal drugs

**Figure 1:**
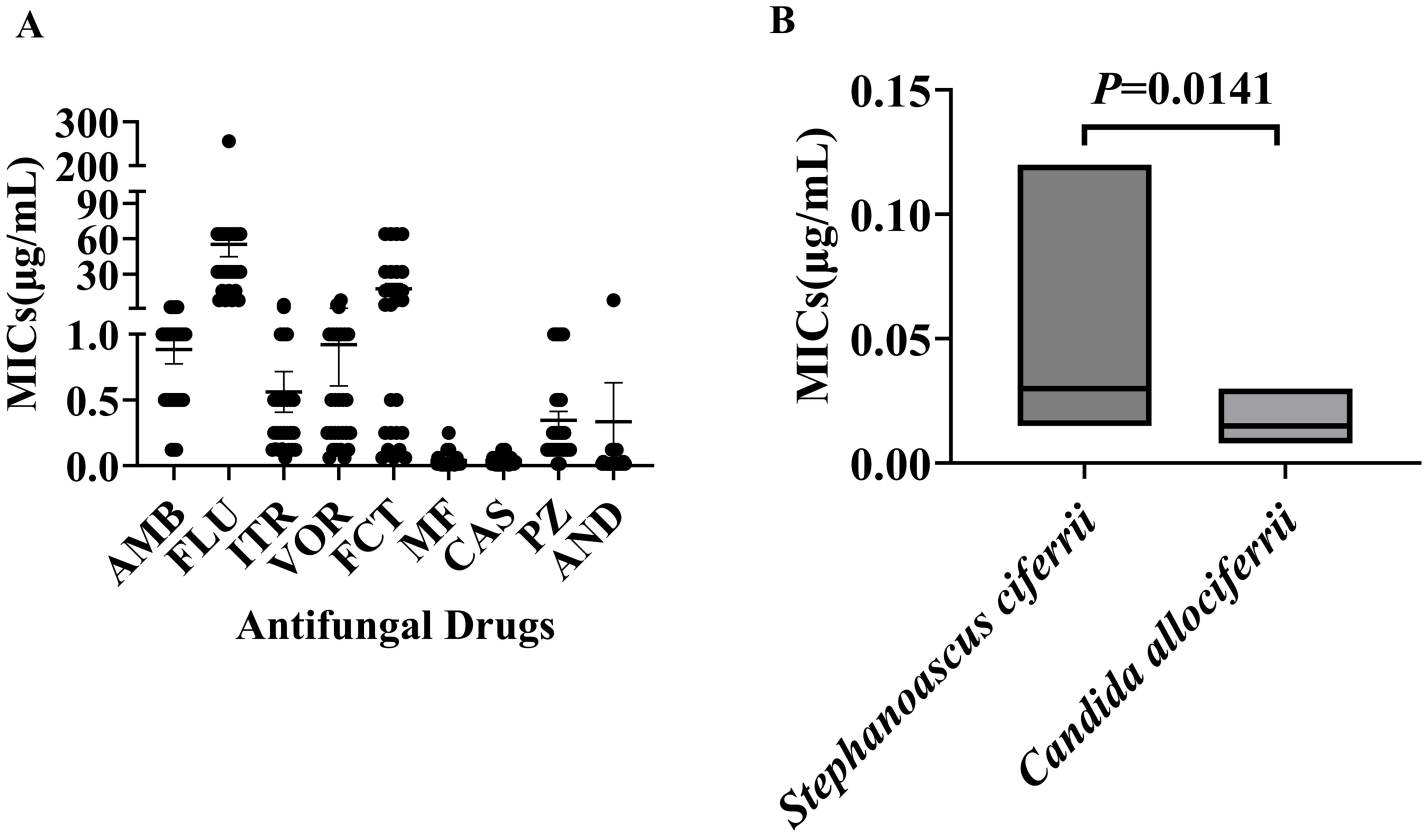
Distribution of minimum inhibitory concentrations of strains of the *Stephanoascus ciferrii* complex against a range of antifungal drugs. **(A)** Antifungal drug susceptibility of the *Stephanoascus ciferrii* complex. **(B)** MICs of *Stephanoascus ciferrii* and *Candida allociferrii* against caspofungin.

The antifungal susceptibility testing results for the 27 strains of *Stephanoascus ciferrii* complex are presented in Figure 1. MIC50 and MIC90 were 1 and 2 μg/mL, respectively, for amphotericin B; 32 and 128 μg/mL, respectively, for fluconazole; 0.25 and 1 μg/mL, respectively, for itraconazole; 0.5 and 1 μg/mL, respectively, voriconazole; 8 and 64 μg/mL, respectively, for 5-fluorocytosine; 0.015 and 0.12 μg/mL, respectively, for micafungin; 0.03 and 0.12 μg/mL, respectively, for caspofungin; 0.25 and 1 μg/mL, respectively, for posaconazole; and 0.015 and 0.12 μg/mL, respectively, for anidulafungin. These results indicated that the *S. ciferrii* complex exhibited high MICs against azoles, particularly fluconazole, while it demonstrated lower MICs against echinocandins. Furthermore, *S. ciferrii* displayed higher MICs against caspofungin than *C. allociferrii* (0.0500 ± 0.0396 vs. 0.0173 ± 0.0092, *P* < 0.05), as shown in Figure 1B. For other antifungal agents, no discernible differences in susceptibility were observed across the three species of the complex.

### 3.3 Biofilm formation ability of the *S. ciferrii* complex

**Figure 2:**
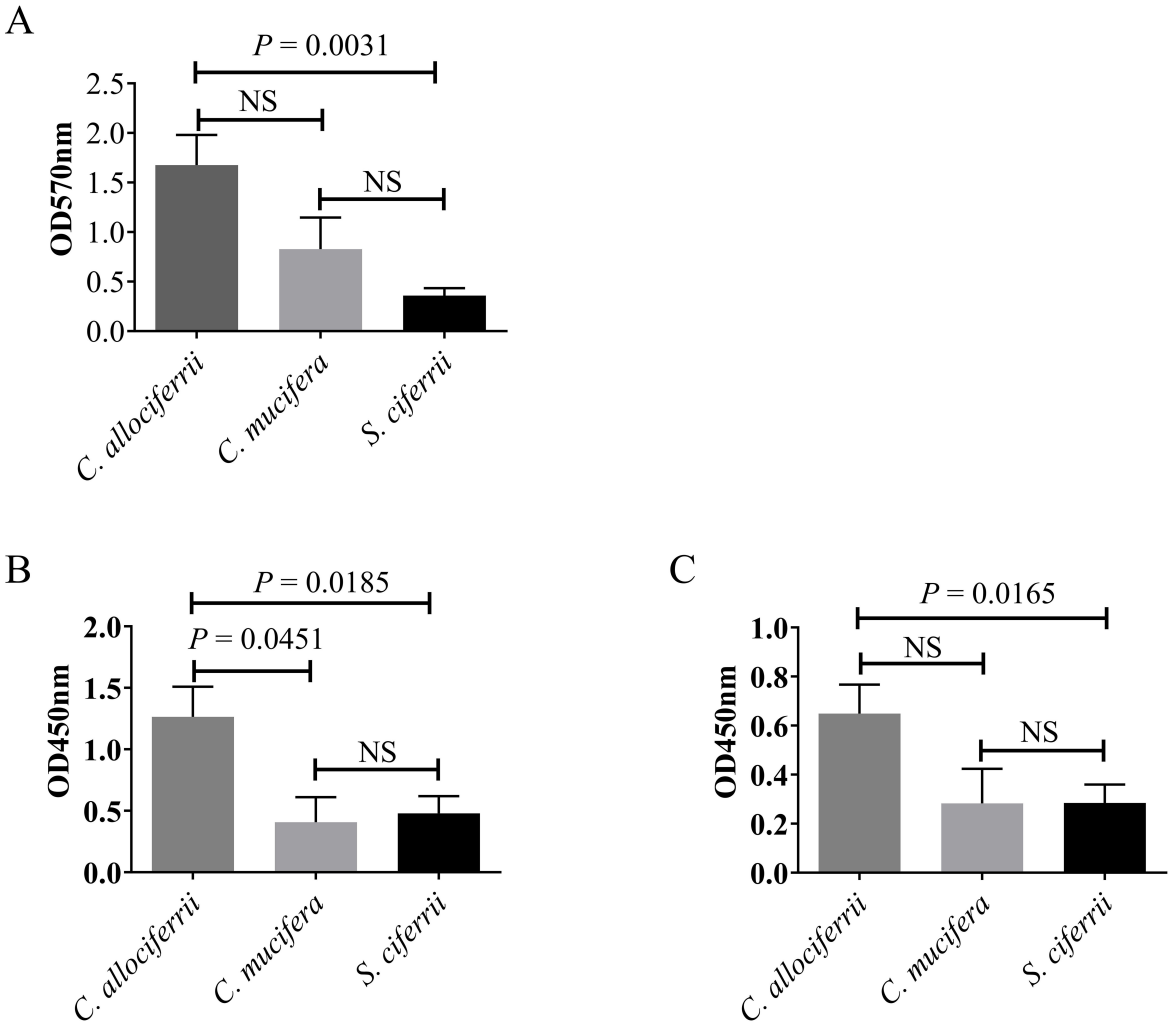
Comparison of biofilm formation between the three species of the *S. ciferrii* complex. **(A)** Biofilm formation ability analyzed by Cystal violet assay in RPMI-1640 medium for 48 h. **(B, C)** Biofilm metabolic activity determined by XTT-assay in RPMI-1640 medium for 48 h **(A)** and 72 h **(B)**, respectively. NS, non-significant difference; OD, optical density.

Assessment of the biofilm formation abilities of the 27 *S. ciferrii* complex strains showed that in the RPMI-1640 medium, *C. allociferrii* demonstrated superior biofilm formation capability to *S. ciferrii* (1.6767 ± 0.6406 vs. 0.3600 ± 0.0816, *P* = 0.0031). No significant difference in biofilm formation was observed between *C. allociferrii* and *C. mucifera* (1.6767 ± 0.6406 vs. 0.8280 ± 0.5060, P > 0.05) or between *S. ciferrii* and *C. mucifera* (0.3600 ± 0.0816 vs. 0.8280 ± 0.5060, *P* > 0.05) (Figure 2). Conversely, in YPD medium, biofilm formation was negligible for all strains, except for one strain that showed minimal development.

Biofilm metabolic activity determined by XTT assay showed that in the RPMI-1640 medium, *C. allociferrii* exhibited elevated biofilm metabolic activity to *S. ciferrii* and *C. mucifera* (*P* = 0.0185 and *P* = 0.0451) after incubated for 48 h. No significant difference in biofilm metabolic activity was detected between *S. ciferrii* and *C. mucifera* (Figure 2). For 72 h, *C. allociferrii* showed higher biofilm metabolic activity to *S. ciferrii* (*P* = 0165). Conversely, in YPD medium, all strains exhibited extremely low biofilm metabolic activity.

### 3.4 Pathogenicity of the *S. ciferrii* complex

**Figure 3:**
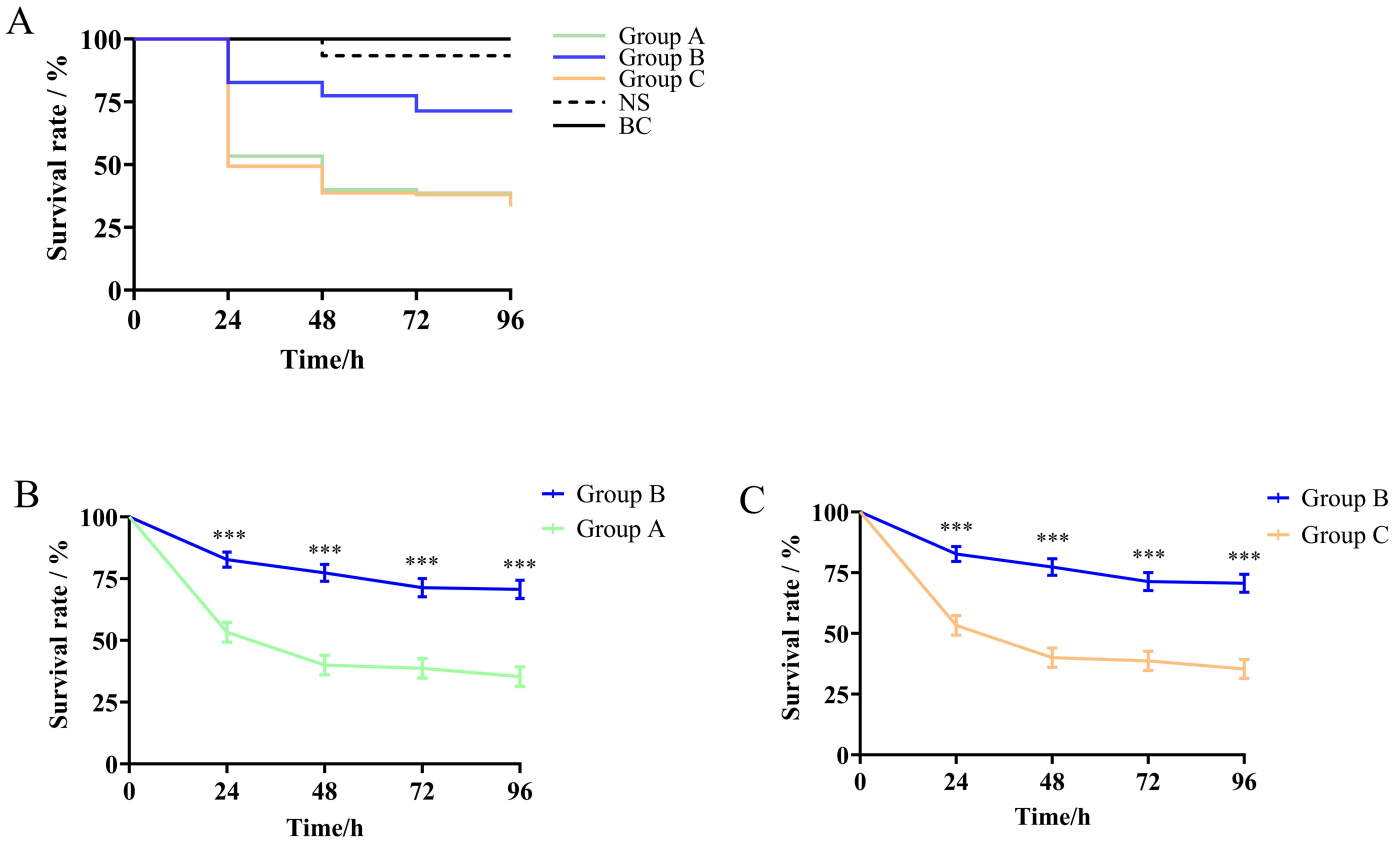
Survival rates of *Galleria mellonella* infection model larvae infected with different species of the *Stephanoascus ciferrii* complex. **(A)** Survival rates of *Galleria mellonella* infection model larvae in different groups. **(B)** The survival rate between *S. ciferrii* and *C. allocofferrii*. **(C)** The survival rate between *S. ciferrii* and *C. mucifera*. Group A, *C. allocofferrii*. Group B, *S. ciferrii*. Group C, *C. mucifera*. BC, blank control; NS, normal saline. ^***^P < 0.001.

In the *G. mellonella* infection model, we adopted a final infection concentration of 1 × 10^6^ CFU/mL and examined the survival rates of larvae infected with different species of the *S. ciferrii* complex. The survival rates of larvae infected with the *S. ciferrii* complex were 60%, 50%, and 48% at 24, 48, and 72 h, respectively. As shown in Figure 3, after 24 h, the survival rates for larvae infected with *S. ciferrii, C. allociferrii*, and *C. mucifera* were 76%, 53%, and 50%, respectively, which declined to 71%, 40%, and 39%, respectively, at 48 h, and further reduced to 65%, 39%, and 39%, respectively, at 72 h. Survival curve analysis showed that the lethality rate of *S. ciferrii* was significantly lower than that of *C. allociferrii* (*P* < 0.0001) and *C. mucifera* (*P* < 0.0001) (Figure 3). In contrast, the survival rate for the blank control group remained at 100%, with the normal saline group's survival rate decreasing to 90% at 72 h.

## 4 Discussion

In this study, we collected 27 clinical strains of the *S. ciferrii* complex that were isolated from patients between 2012 and 2023, in order to explore and compare their morphology, antifungal drug susceptibility, biofilm formation ability, and fatality rate in *G. mellonella* larvae as an animal infection model were performed. This is the first study to comprehensively examine these characteristics of the complex, and we believe that the data make an important contribution to the current literature while also laying the foundation for future *in vitro* and *in vivo* investigations that could potentially be translated to the clinical context.

In the first set of experiments, we compared the ability of different methods to identify 27 *S. ciferrii* complex strains. With ITS sequencing, 27 clinical strains of the *S. ciferrii* complex were classified as 15 strains of *S. ciferrii* (55.56%), 7 strains of *C. allociferrii* (25.93%), and 5 strains of *C. mucifera*. While the VITEK 2 Compact system detected all 27 strains, they were all identified as *S. ciferrii*. In contrast, only 14 strains were detected by mass spectrometry, which successfully identified 80% (12/15) of *S. ciferrii* strains, 14.29% (1/7) of *C. allociferrii* strains, and 20% (1/5) of *C. mucifera* strains. Thus, neither the mass spectrometry nor the biochemical identification technology was able to achieve accurate identification of the *S. ciferrii* complex. This observation is consistent with that of Guo et al. (Guo et al., 2021). The scarcity of reference data for *C. allociferrii* and *C. mucifera* may have hindered the identification capabilities of MS, emphasizing the pressing need to delve into the species-level characteristics of the *S. ciferrii* complex to add more comprehensive reference data and to advance identification technologies. The biochemical method (VITEK 2) also faced similar limitations in terms of the lack of comprehensive databases within existing commercial systems. Another limitation to the identification of strains within this complex is the high degree of similarity in their morphological characteristics, as no noticeable distinctions were observed between members of the complex. Thus, accurate identification of strains of the *S. ciferrii* complex is reliant on molecular-level detection methods that need to be advanced further through more characterization studies in the future. Finally, according to the ITS identification results, *S. ciferrii* had the highest isolation rate, which partly reflects its higher prevalence within the complex and also points to the need to focus on detection of this species. Further studies can help verify and further expand on the identification of species that more prevalent and need more attention in the diagnosis of fungal infections caused by the *S. ciferrii* complex.

The antifungal susceptibility test results indicated that the *S. ciferrii* complex exhibited low MICs against echinocandins, which makes these agents suitable options for treating *S. ciferrii* complex infections. In contrast, the MICs for azoles—specifically posaconazole, voriconazole, and itraconazole—were significantly higher than those for echinocandins or amphotericin B. Notably, extensively used of broad-spectrum antimicrobial agent itraconazole led to a significant proportion of the *S. ciferrii* complex displayed resistance to itraconazole, in 54% of skin and skin appendage disorders (de Gentile et al., 1995). Similarity, itraconazole resistance was also detected in 41% of bloodstream infections (Sathi et al., 2022). On the other hand, echinocandins have a broad spectrum of activity that includes inhibition of β-(1,3)-glucan synthesis in the fungal cell wall (Cosio et al., 2024). A noteworthy finding that emerged was that all strains exhibited high MICs against fluconazole that exceeded 32 μg/mL, while a subset exhibited MICs of 8 μg/mL or 16 μg/mL. This is consistent with the study of Antonio Pérez-Hansen (Pérez-Hansen et al., 2019), which reported a significantly high MIC of 32 μg/mL for fluconazole against the *S. ciferrii* complex. In contrast to our observations, Hasan Agin reported a case of systemic mycosis in which a patient was infected by the *S. ciferrii* complex, which exhibited high resistance to fluconazole, amphotericin B, caspofungin, and anidulafungin (Agin et al., 2011). This variability in resistance patterns may arise from strain-dependent differences or external environmental factors (Cosio et al., 2024). For example, in the present study, *S. ciferrii* and *C. allociferrii* demonstrated greater resistance to fluconazole than *C. mucifera*. Fluconazole is a first-line triazole antifungal agent that inhibits fungal cytochrome P450 activity, thereby reducing ergosterol synthesis and compromising cell membrane integrity (Cheng et al., 2004). Several studies have investigated the genetic basis for resistance against fluconazole. In one such study, Sasani et al. established a link between biofilm formation and overexpression of the *ERG11* and *UPC2* genes in *Candida tropicalis* that was associated with fluconazole resistance (Sasani et al., 2021b). Further, Abbes et al. demonstrated that overexpression of the *Mdr* gene in *Trichosporon asahii* isolates was significantly correlated with fluconazole resistance (Abbes et al., 2021). These findings imply that fluconazole resistance may have a genetic basis that warrants further investigation. Species-dependent differences were also observed for the antifungal agent caspofungin, as *S. ciferrii* displayed higher MICs than *C. allociferrii* (0.0500 ± 0.0396 vs. 0.0173 ± 0.0092, *P* = 0.0141) (Figure 1B). However, for other antifungal agents, no significant species-level differences were observed among the three species of the *S. ciferrii* complex. Despite the variations observed, the findings do provide valuable *in vitro* evidence for the antifungal capabilities of various agents against infections caused by the *S. ciferrii* complex.

When evaluating the antifungal abilities of agents and resistance to treatment, non-molecular virulence factors also need to be considered, for example, biofilm formation, environmental factors, and selective pressure resulting from prior exposure to antifungals. The emergence of echinocandin-resistant *C. albicans* and *C. glabrata* in Switzerland highlights the complexities of antifungal resistance mechanisms (Coste et al., 2020) and underscores the significant public health challenges in treating fungal infections and managing antifungal resistance effectively (Paiva and Dias, 2024). However, the *S. ciferrii* complex has been overlooked in previous research, and this limits our understanding of the fluconazole resistance mechanisms of these species. Furthermore, the lack of genomic data hinders investigations into the gene expression patterns of this complex (Mixão et al., 2019). Again, this reiterates the need for more in-depth molecular and genomic studies into this fungal complex.

Biofilms, which are protective structures composed of microbial cells and extracellular matrix, can adhere to both abiotic and biotic surfaces (Flemming et al., 2023). Biofilms facilitate microbial adherence to medical devices, skin, and mucosal membranes, potentially leading to persistent nosocomial infections (Tanu et al., 2021; Hall and Mah, 2017). This makes biofilm formation ability an important indicator of the pathogenicity of an organism. Accordingly, we investigated the biofilm formation ability of the *S. ciferrii* complex and found that it was highly influenced by the growth medium. All three species formed biofilms in the RPMI-1640 medium. In contrast, in the YPD medium, all strains displayed minimal biofilm formation, with only one strain showing marginal development of biofilms. Meanwhile, the XTT-reduction assay detected low biofilm metabolic activity following incubation in YPD medium. This indicates that environmental factors significantly affect the biofilm formation ability of *S. ciferrii* complex strains. Remarkably, in the RPMI-1640 medium, *C. allociferrii* displayed the highest biofilm formation rate; it was followed by *C. mucifera*, with *S. ciferrii* exhibiting the lowest biofilm formation rate. Additionally, in RPMI 1640 medium, the XTT assay results further demonstrated that the biofilm metabolic activity of the strains correlated well with their biofilm formation ability. This phenomenon may be related to the nutrient utilization characteristics of each species. Accordingly, Chakraborty indicated that nutrient conditions affect pathogen growth, morphology, and biofilm formation (Ramesh et al., 2023). According to the epidemiological data at our hospital, infections attributed to the *S. ciferrii* complex predominantly manifest as superficial infections of the ear canal. Therefore, we speculate that the distinctive features of the ear canal environment, particularly its oily characteristics, may be related with infection onset and chronic infections of the *S. ciferrii* complex. However, the correlation between the ear canal environment and its propensity for bacterial and fungal colonization and infection remain unclear and require further research. Investigating the related mechanisms could help in the development of new strategies to deal with biofilm formation by the *S. ciferrii* complex.

*G. mellonella* larvae serve as an effective infection model for assessing pathogenicity among different species due to its similarities in immune response with mammals and ease of culture and inoculation (Cotter et al., 2000). Through our preliminary experiments with a range of concentrations for inducing infection, from 1 × 10^7^ CFU/mL to 1 × 10^3^ CFU/mL, we found that 1 × 10^6^ CFU/mL was the ideal concentration to establish the infection assay in this model. The larvae were first divided into three groups representing the three different species of the *S. ciferrii* complex, with each group subsequently subdivided into five subgroups that received yeast suspensions derived from distinct strains within the same species, with a total of 15 strains were included in the experimental study. *C. allociferrii* and *C. mucifera* had higher lethality rates than *S. ciferrii* in the *G. mellonella* larva infection model. As the culture duration increased, the survival rates of larvae infected with all *S. ciferrii* complex species gradually declined before they stabilized, with the survival rates of larvae infected with *C. allociferrii* and *C. mucifera* being notably lower than the survival rates of larvae infected with *S. ciferrii*. These findings indicate that *C. allociferrii* and *C. mucifera* had stronger pathogenicity and need to be considered when testing for this complex in the clinical setting.

The pathogenicity exhibited by the three species of the *S. ciferrii* complex correlated with their biofilm formation capabilities, although their antifungal resistance profiles differed. That is, *S. ciferrii* exhibited limited capacity for biofilm formation and weak virulence, yet displayed notable resistance to antifungal agents such as fluconazole. Conversely, *C. allociferrii* and *C. mucifera* exhibited higher levels of biofilm formation and stronger pathogenicity, but were more sensitive to antifungal treatments. Studies have shown that enhanced biofilm formation capabilities in microorganisms, such as *C. albicans*, are associated with increased pathogenicity *in vivo* (Xiong et al., 2024). Further, research indicates that *C. auris* possessing a non-aggregate growth phenotype possesses superior biofilm-forming capacity to strains that aggregate due to division anomalies, and at the same time, it is more likely to cause disseminated infection and has stronger pathogenic properties in *G. mellonella* larvae. However, it has also been demonstrated that nonaggregative *C. auris*, resulting from different cell-to-cell adhesion capacity, exhibit greater capacity to form biofilms and enhanced virulence capacity (Sherry et al., 2017). With regard to the relationship between pathogenicity and treatment sensitivity, *C. auris* demonstrate low sensitivity against antifungal agent, leading to unexpectable outbreaks in clinical recently.

This study is limited by the small number of strains analyzed, particularly *C. allociferrii* and *C. mucifera*. Thus, future research should aim to include a broader range of samples to validate the study's findings. Due to the lack of existing genomic data about this complex, this study failed to explore the molecular mechanisms of drug resistance, biofilm formation, and virulence.

## 5 Conclusions

In summary, our study reveals for the first time the pathogenic discrepancies and biofilm formation differences among the three known species that form the *S. ciferrii* complex. Initially, we discovered that *S. ciferrii* has the highest prevalence in the complex by ITS sequencing. However, none of the commonly used clinical testing methods (morphological identification, biochemical reaction assay, and MS identification) were able to identify *C. allociferrii* and *C. mucifera* species in the complex. This points to the urgent need to improve the current techniques for rapid identification of the *S. ciferrii* complex. Second, the antimicrobial susceptibility data, biofilm formation capacity, and *G. mellonella* larva infection model demonstrated that *S. ciferrii* exhibited a significantly higher MIC for fluconazole than the two other species, with a lower biofilm formation capacity and lower mortality rate than *C. allociferrii*. Thus, this study reveals the differences in pathogenic characteristics among the three species of the *S. ciferrii* complex and provides an important theoretical basis for further in-depth study of the clinical pathogenic characteristics of each species in the complex and the search for treatment measures. Importantly, the paucity of literature and data, coupled with the rising incidence of *S. ciferrii* infections, indicates a pressing need for heightened attention to this organism.

## Data Availability

The original contributions presented in the study are included in the article/Supplementary material, further inquiries can be directed to the corresponding author.
