# Supplementary material for: Clinical isolation, biofilm formation, and pathogenicity analysis of different species of the Stephanoascus ciferrii complex
Source: Front Microbiol. 2025 Apr 9;16:1570952. doi: 10.3389/fmicb.2025.1570952 (PMC12014652; doi:10.3389/fmicb.2025.1570952)
Supplement: Supplementary file 1 [file Data_Sheet_1.docx]

Supplementary Table 1. Antifungal drug susceptibility of the *Stephanoascus* ciferrii complex.

|  | Species | AMB | FLU | ITR | VOR | FCT | MF | CAS | PZ | AND |
| --- | --- | --- | --- | --- | --- | --- | --- | --- | --- | --- |
| S-1 | *Trichomonascus ciferrii* | 0.5 | 8 | 0.5 | 1 | 16 | 0.015 | 0.03 | 0.015 | <0.015 |
| S-2 | *Candida mucifera* | 0.5 | 16 | 0.25 | 0.12 | <=0.06 | <=0.008 | <=0.008 | 0.12 | <=0.015 |
| S-3 | *Trichomonascus ciferrii* | 1 | 32 | 0.12 | 0.25 | 0.12 | 0.12 | 0.12 | 0.12 | 0.12 |
| S-4 | *Candida allociferrii* | 0.5 | 8 | 0.12 | 0.06 | 0.12 | <=0.008 | 0.015 | 0.25 | <=0.015 |
| S-5 | *Candida allociferrii* | 0.5 | 64 | 0.5 | 1 | 32 | 0.015 | 0.03 | 0.12 | <=0.015 |
| S-6 | *Trichomonascus ciferrii* | 1 | 128 | 4 | 4 | 32 | 0.015 | 0.015 | 0.12 | <=0.015 |
| S-7 | *Candida mucifera* | <=0.12 | 64 | 0.125 | 0.06 | 4 | <=0.008 | <=0.008 | 0.015 | <=0.015 |
| S-8 | *Trichomonascus ciferrii* | 0.5 | 32 | 0.25 | 0.25 | 16 | 0.015 | 0.03 | 0.25 | <=0.015 |
| S-9 | *Candida allociferrii* | 1 | 64 | 2 | 1 | 0.25 | 0.015 | 0.015 | 0.5 | <=0.015 |
| S-10 | *Candida mucifera* | 2 | 64 | 0.5 | 0.5 | 8 | 0.25 | 0.12 | 1 | 8 |
| S-11 | *Trichomonascus ciferrii* | 2 | 256 | 1 | 8 | 64 | 0.12 | 0.06 | 1 | 0.12 |
| S-12 | *Trichomonascus ciferrii* | 2 | 128 | 0.5 | 1 | 64 | 0.12 | 0.12 | 1 | 0.12 |
| S-13 | *Trichomonascus ciferrii* | 1 | 64 | 0.5 | 0.5 | 64 | 0.06 | 0.06 | 1 | 0.12 |
| S-14 | *Trichomonascus ciferrii* | 1 | 32 | 0.12 | 0.25 | 32 | 0.015 | 0.015 | 0.25 | <=0.015 |
| S-15 | *Candida allociferrii* | 1 | 32 | 0.25 | 0.25 | 0.25 | 0.015 | 0.03 | 0.5 | <=0.015 |
| S-16 | *Candida mucifera* | 1 | 8 | 0.12 | 0.12 | 0.25 | 0.06 | 0.06 | 0.12 | 0.12 |
| S-17 | *Candida mucifera* | 2 | 32 | 0.06 | 0.25 | 64 | 0.015 | 0.03 | 0.12 | <=0.015 |
| S-18 | *Trichomonascus ciferrii* | <=0.12 | 64 | 0.5 | 0.5 | 4 | 0.015 | 0.03 | 0.12 | 0.03 |
| S-19 | *Trichomonascus ciferrii* | <=0.12 | 32 | 0.25 | 0.5 | 16 | <=0.008 | 0.015 | 0.12 | <=0.015 |
| S-20 | *Trichomonascus ciferrii* | 1 | 128 | 1 | 1 | 0.5 | <=0.008 | 0.06 | 0.25 | <=0.015 |
| S-21 | *Candida allociferrii* | 1 | 16 | 0.25 | 0.25 | 0.25 | 0.015 | <=0.008 | 0.5 | <=0.015 |
| S-22 | *Trichomonascus ciferrii* | 0.5 | 8 | 0.12 | 0.12 | 8 | 0.015 | 0.03 | 0.12 | <=0.015 |
| S-23 | *Candida allociferrii* | 0.5 | 32 | 0.25 | 0.12 | <=0.06 | 0.015 | 0.015 | 0.25 | <=0.015 |
| S-24 | *Trichomonascus ciferrii* | 1 | 16 | 0.25 | 0.25 | 16 | 0.015 | 0.03 | 0.25 | <=0.015 |
| S-25 | *Candida allociferrii* | 0.5 | 32 | 0.5 | 0.5 | <=0.06 | 0.015 | <=0.008 | 1 | <=0.015 |
| S-26 | *Trichomonascus ciferrii* | 0.5 | 64 | 1 | 2 | 32 | 0.015 | 0.015 | 0.12 | 0.03 |
| S-27 | *Trichomonascus ciferrii* | 1 | 64 | 0.125 | 1 | 0.5 | 0.12 | 0.12 | 0.12 | 0.12 |

Supplementary Table 1. Antifungal drug susceptibility of the *Stephanoascus* ciferrii complex. AMB, Amphotericin B. FLU, Fluconazole. ITR, Itraconazole. VOR, Voriconazole. FCT, Flucytosine. MF, Micafungin. CAS, Caspofungin. PZ, Posaconazole. AND, Anidulafungin.
